# Significance of peritoneal washing cytology in the accurate staging of malignant ovarian tumors

**DOI:** 10.12669/pjms.38.1.4393

**Published:** 2022

**Authors:** Rubina Gulzar, Ruqaiya Shahid, Shazia Mumtaz, Jahan Ara Hassan

**Affiliations:** 1 Dr. Rubina Gulzar, FCPS. Associate Professor, Department of Pathology, Dow International Medical College, Dow University of Health Sciences (DUHS), Karachi, Pakistan; 2 Dr. Ruqaiya Shahid, FCPS. Associate Professor, Department of Pathology, Dow International Medical College, Dow University of Health Sciences (DUHS), Karachi, Pakistan; 3 Dr. Shazia Mumtaz, FCPS. Assistant Professor, Department of Pathology, Dow International Medical College, Dow University of Health Sciences (DUHS), Karachi, Pakistan; 4 Dr. Jahan Ara Hasan, FCPS, Ph.D. Professor, and Head of the Department, Gynecology and Obstetrics, Dow University Hospital, Dow University of Health Sciences (DUHS), Karachi, Pakistan

**Keywords:** Cytology, Ovarian neoplasms, Omentum, Lymphatic metastasis, Peritoneal lavage, Prognosis

## Abstract

**Objectives:**

To identify the percentage of ovarian cancers with positive peritoneal cytology and to correlate the positive cytology with the prognostic factors.

**Methods:**

This retrospective, cross-sectional study, evaluated the data of surgical specimens of malignant ovarian tumors, received in the Department of Pathology, Dow University of Health Sciences over a period of three years. The peritoneal cytology was correlated with these prognostic parameters: the size of the tumor, stage, capsular invasion, omental, and lymph node metastasis.

**Results:**

Eighty malignant ovarian tumors were diagnosed. Serous carcinoma was the most common ovarian tumor, diagnosed in 24(30.0%) cases, followed by endometrioid carcinoma in 17(21.25%) and Granulosa cell tumor in 11 (13.75%) cases. The mean age of the patients was 41.91 years (range 7-71 years). The mean size of the tumors was 10.03 cm (SD 5.62 cm). The ovarian capsular invasion was present in 27(33.75%) tumors. Peritoneal cytology was positive in 10/24 cases, with a detection rate of 41.66%. Omentum was involved in 12/34(35.29%) cases. Lymph node dissection was performed in three cases, two were reported as positive for metastasis. Peritoneal cytology significantly correlated with the tumor size (p=0.045), and with ovarian capsular invasion (p=0.054) and omental metastasis (p=0.052). Most of the tumors were staged as FIGO stage IA.

**Conclusion:**

Peritoneal cytology correlates with the tumor size, stage, and omental metastasis of the malignant ovarian tumors. It should be routinely performed at the time of surgery for the optimal staging of the patients.

## INTRODUCTION

Ovarian cancer is one of the leading causes of motility and morbidity throughout the world.^1^ Ovarian cancer has an age-standardized incidence rate (ASR) of 7/100,000 and a mortality rate of 3.8/100,000 females in the world, according to Globocan 2018.^1^ In Pakistan ovarian cancer is the seventh common cancer in Pakistan, with an ASR of 3.3/100,000 female population, which is lower than the neighboring countries like Turkey (6.3), Afghanistan (3.8), China (4.1), and India (4.9).^2^

Ovarian cancer is a silent killer for females; as ovaries are retroperitoneal organs, the symptoms of these tumors are vague, and most of the patients present at a late stage, with dissemination into the abdominal cavity and omentum.^3^

The staging of ovarian tumors depends upon the capsular invasion and on the trans-coelomic spread of the disease.^1^ Peritoneal washing is taken at the time of surgery, and the fluid is evaluated for the presence of malignant cells.^4^,^5^ Positive peritoneal cytology is associated with morbidity and tumor recurrence.^4^ According to the revised staging by the International Federation of Gynecology and Obstetrics (FIGO), positive peritoneal cytology upstages the ovarian cancer from IA to IC, which requires chemotherapy after the surgery.^4^,^6^ The absence of peritoneal cytology with the surgical specimen results in an incomplete staging of the ovarian cancers. This study was conducted to highlight the importance of peritoneal cytology in the accurate staging of malignant ovarian tumors. The objective of this study was to identify the percentage of ovarian cancers with positive peritoneal cytology and to correlate the positive cytology with the prognostic factors.

## METHODS

This retrospective, cross-sectional study was conducted in the Department of Histopathology, Dow University of Health Sciences. Approval from the Institutional Review Board was obtained (IRB-1270/DUHS/Approval/2019). Cases were retrieved from the archives (compiled hard copies of reports) from 1^st^ Jan 2017 till 31^st^ Dec 2019. Excision specimens of malignant ovarian tumors were included in the study. Benign and borderline ovarian tumors and core biopsies were excluded. The parameters recorded on a predesigned Performa were: age of the patient, surgical procedure, histopathological classification of the tumor, size, capsular integrity, omentum and lymph node metastasis, peritoneal washing cytology diagnosis, and stage. Tumor histopathological classification was according to the World Health Organization (WHO) and staging was according to the FIGO protocols.^4^,^7^

The protocol for pelvic fluid aspiration was: The abdominal cavity is opened, before approaching any viscera (to avoid spill-over), 100ml of normal saline is inserted within the pelvic cavity, and after an interval of 5-10 minutes, all the fluid is aspirated. The obtained peritoneal fluid is transported to the histopathology lab, where it is centrifuged. The extracted material is used to prepare four smear slides which are immediately fixed with 95% alcohol. The remaining material is used for cell block preparation. Smear and cell block are stained with Hematoxylin and Eosin for microscopic examination. Immunohistochemical staining (IHC) may be performed on the cell blocks, in cases that require confirmation of diagnosis. The most commonly used stains in our lab are Calretinin, Ber-Ep4, WT1, P53, and Cytokeratins 7 and 20 (DAKO Envision automated system). A cytologist and a consultant pathologist report the cases of peritoneal cytology and they routinely consult the ovarian histopathological diagnosis and may view the slides.

Data were analyzed in Statistical Package for the Social Sciences (SPSS, IBM, version 20). Means were calculated for the age of the patient and for the size of the tumor. Two categories were created for the size of tumors; ≥10 cm and <10 cm. For correlation of peritoneal cytology with the prognostic parameters, Chi-square and Fisher exact tests were used, with a confidence interval of 95% and a level of significance of 0.05.

## RESULTS

Total 80 malignant ovarian tumors were diagnosed in three years. The mean age of patients was 41.91 years (±12.60 SD, range 7-71 years). The histopathological diagnosis of the ovarian tumors is provided in Table-I.

**Table I:** Histopathological classification of the malignant ovarian tumors (N=80).

Malignant ovarian tumors	Frequency	Percentage %
1. Surface epithelial tumors	57	71.25 %
Serous carcinoma	24	30.00 %
Endometrioid carcinoma	17	21.25 %
Clear cell carcinoma	6	7.50 %
Mucinous carcinoma	5	6.25 %
Seromucinous carcinoma	3	3.75 %
Carcinosarcoma	2	2.50 %
2. Germ cell tumors	4	5.00 %
Dysgerminoma	3	3.75 %
Yolk sac tumor	1	1.25 %
3. Sex cord stromal tumors	13	16.25 %
Granulosa cell tumor	11	13.75 %
Sertoli-Leydig cell tumor	2	2.50 %
4. Metastatic tumors	6	7.50 %
Colon, appendix	2	2.50 %
Gastric	3	3.75 %
Breast	1	1.25 %

Total	80	100.0 %

The prognostic factors of the malignant ovarian tumors were: mean size was 10.03 cm (SD 5.62 cm), capsular invasion was present in 27(33.75%) tumors. Omentum was received in 34 patients, and was positive for metastasis in 12/34(35.29%) cases. Lymph node dissection was performed in only three cases, two of these were positive for metastasis. Peritoneal cytology was received in only 24(30%) cases, and was positive for malignant cells in 10/24(41.66%) cases. The correlation of peritoneal cytology with the prognostic factors is provided in Table-II.

**Table II:** Correlation of peritoneal cytology with the prognostic factors of malignant ovarian tumors.

Peritoneal cytology	Tumor size<10cm	Tumor size>or equal to10cm	Total N (%)	p-value; test applied
Positive	7 (29.16%)	3 (12.50%)	10 (41.66%)	0.045; Chi square
Negative	4 (16.66%)	10 (41.66%)	14 (58.33%)	
Total	11 (45.83%)	13 (54.16%)	24 (100%)	

Peritoneal cytology	Ovarian surface involvement present	Ovarian surface involvement absent	Total	

Positive	6 (25.0%)	4 (16.66%)	10 (41.66%)	0.054; Chi square
Negative	3 (12.50%)	11 (45.83%)	14 (58.33%)	
Total	9 (37.50%)	15 (62.50%)	24 (100%)	

Peritoneal cytology	Omentum involved	Omentum not involved	Total	

Positive	3 (23.07%)	1 (7.69%)	4 (30.77%)	0.052; Fisher exact
Negative	1 (7.69%)	8 (61.53%)	9 (69.23%)	
Total	4 (30.77%)	9 (69.23%)	13 (100%)	

Peritoneal cytology was not associated with age category (p=0.134) and stage of tumor (p=0.272). No test could be computed for an association of peritoneal cytology with the histologic type of tumor and lymph node status due to the small sample size.

Total abdominal hysterectomy and bilateral salphigo-oophorectomy (TAH&BSO) was the most common surgical procedure performed in 44(55.0%) patients, followed by Oophorectomy in 36(45.0%).

**Fig.1:**
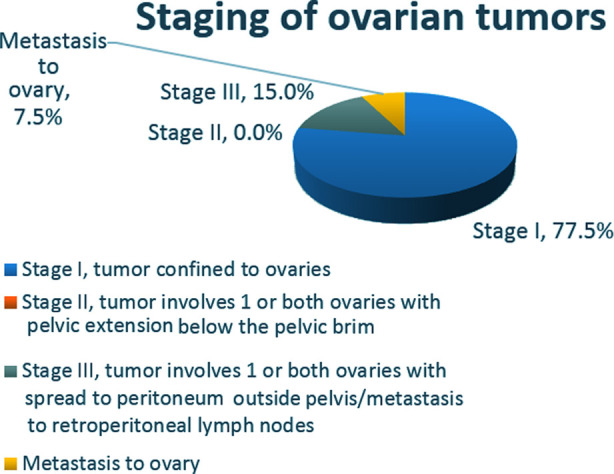
FIGO cancer stage for malignant ovarian tumors.

FIGO stage classification of ovarian cancers was performed in all cases (Fig.1). Sixty-two cases (77.50%) were stage I, of these 39(48.75%) were stage IA, where the tumor is confined to ovaries and fallopian tubes, the rest were stage IC (Fig.1).

## DISCUSSION

The results of our study conclude that the surface epithelial tumors are the most common type of malignant ovarian tumors. Of these, the serous carcinomas are the most frequent. Serous carcinomas have been reported as the most common type of malignant tumors in previous studies from Pakistan, India, and Bangladesh; however, all these studies have reported mucinous carcinoma to be the second common cancer, in contrast to endometrioid carcinoma in our study.^8^-^10^ Globally, ovarian cancer has a high incidence in European descent, and Serous carcinoma is the most common malignant ovarian tumor in Europe and the USA. In contrast, Japan, Thailand, and Singapore report a high percentage of Endometrioid, Mucinous, and Clear cell carcinomas.^11^

**Fig.2:**
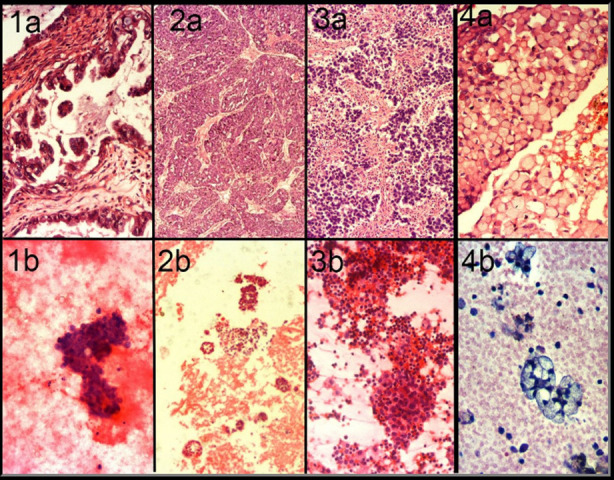
Malignant ovarian tumors; a=histology, b=cytology 1: Serous carcinoma, 2: Endometrioid carcinoma, 3: Granulosa cell tumor. 4: Metastatic carcinoma to the ovary.

The positive rate for peritoneal cytology in the current study was 41.6%. Detection rates of malignant cells in peritoneal cytology are 25% by Fadare et al,^12^ 62.2% by Ozkara et al^13^ and 20% by Sanchs et al.^14^ Naz et al.^8^ reported a detection rate of 76.9% for serous carcinoma, 44 % for endometrioid, and 25% for mucinous carcinoma. Variation in rates may be due to the technique of obtaining cytology, irregular exfoliation of different ovarian tumors, differences in cytopathologist opinion.^8^ Another limitation of cytologic examination is false-positive and false-negative results. A false-positive result may be seen in endometriosis, endosalpingiosis, and reactive mesothelial cells; whereas, low cellularity may result in false-negative cases.^15^ A benign condition may be favored in the presence of cilia in the cells, no single cells, few cells with cytoplasmic vacuoles, absence of mitotic activity, and two distinct populations not identified.^16^ Use of IHC stains, as in our study, has been reported to increase the diagnostic accuracy of peritoneal washing.^17^ Serous ovarian tumors are characterized by small, poorly cohesive papillary fragments in cytology; whereas, endometrioid and mucinous carcinomas show features of adenocarcinoma (Fig-2). Metastatic carcinoma shows mucin and signet ring cells (Fig.2). Correlation with the history and ovarian biopsy helps in deducing a conclusion.

Peritoneal cytology in the current study significantly correlated with the tumor size, here the small size of ovarian tumors were associated with positive cytology for malignant cells. It also correlated with ovarian surface involvement and omentum status. These findings are in concordance with those of Naz et al. who have reported a correlation of peritoneal cytology with tumor size, omental metastasis, and capsular invasion.^8^ Ozkara et al. have reported a positive correlation of cytology with the grade, stage, lymph node involvement, and bilaterality of the ovarian carcinoma.^13^

Most of the tumors were FIGO stage IA on the available biopsy material (Fig.1). This may not be a true representation of staging, as staging laparotomy was not performed in most of our cases. In case of a pre-operative diagnosis of ovarian malignancy, staging laparotomy is advocated by FIGO, which includes surgical evaluation of all peritoneal surfaces, peritoneal washing or retrieval of ascites, infra-colic omentectomy and selective lymphadenectomy of pelvic and para-aortic lymph nodes, TAH BSO in most cases, and appendectomy in mucinous tumors and biopsy of any suspicious lesion.^4^ In young patients with stage I cancer, wishing to preserve fertility, preservation of contralateral ovary and uterus is recommended.^4^ Germ cell tumors are highly sensitive to platinum-based chemotherapy therefore, conservative surgery is recommended.^4^ For pathology, a thorough examination of the fallopian tubes and the ovarian capsule is recommended.^4^ We suggest that a complete clinical and radiological workup should be performed for a preliminary diagnosis, for clinical staging, and for the planning of surgery appropriate for that stage.

### Strength and Limitations of the study:

The strength of this study was that the histopathologist and the cytopathologist routinely consulted with each other and IHC was employed for a definite diagnosis. The limitations of the study were small sample size, a restricted period of the study; unavailability of clinical and radiological information to the pathologist in most cases.

## CONCLUSION

We conclude that the peritoneal washing cytology is an important tool in the staging of ovarian tumors and should be acquired in every suspected malignant ovarian tumor.

## What will the study add to the literature:

This study emphasizes on the importance of following FIGO guidelines and performing staging laparotomy in malignant ovarian tumors. Not only peritoneal cytology but lymph node dissection and omentectomy have role in surgical staging, thus prognosis of the patients.
